# The study on the clinical effectiveness and safety of traditional Chinese medicine acupoint catgut embedding guided by musculoskeletal ultrasound in the treatment of nerve root sciatica: A protocol for systematic review and meta-analysis of randomized controlled trials

**DOI:** 10.1097/MD.0000000000025387

**Published:** 2021-04-02

**Authors:** Zhaoyi Han, Xiaowei Li, Zhi Liu, Morigen Bai, Zhihui Zhao, Junqing Wang

**Affiliations:** a Department of Ultrasound; b Department of Anesthesiology, Inner Mongolia Baogang Hospital (The Third Affiliated Hospital of Inner Mongolia Medical University), No. 20 Shaoxian Road, Kundulun District, Baotou 014010, Inner Mongolia Autonomous Region, China.

**Keywords:** acupoint catgut embedding, acupuncture, musculoskeletal ultrasound, nerve root sciatica, randomized controlled trials, systematic review and meta-analysis, traditional Chinese medicine

## Abstract

**Background:**

Nerve root sciatica (NRS) is a common orthopedic disease, which usually occurs between 20 and 40 years of age, and the incidence rate is increasing year by year and is being younger. The disease has no special effect of treatment, clinically generally taking the symptomatic treatment, such as taking short-term glucocorticoids, sedatives, analgesics, and so on. Long-term use of drugs will adversely affect the patient's gastrointestinal tract, liver, and kidney function. The surgical treatment has a high risk of surgery, high cost, side effects, and other problems, so the choice of treatment method has always been a difficult problem in clinical and scientific research. The study shows that 90% of patients with sciatica can be cured by non-surgical treatment, so conservative therapy is often used in the treatment of sciatica, traditional Chinese medicine treatment methods in the treatment of NRS has been widely used, which has achieved good results, but there is no evidence of evidence-based medicine. Therefore, this study uses systematic evaluation to conduct the scientific evaluation of the clinical effectiveness and safety of traditional Chinese medicine acupoint catgut embedding guided by musculoskeletal ultrasound in the treatment of NRS, and provide evidence-based medical evidence support for the treatment of NRS.

**Methods:**

Using the computer to retrieve the PubMed, ScienceDirect, Web of Science, Embase, Cochrane Library, CNKI, VIP, WANFANG Database, and CBM. Using the subject words and terminology words to retrieve the Chinese-English database and retrieve a randomized controlled study on the clinical effectiveness and safety of traditional Chinese medicine acupoint catgut embedding guided by musculoskeletal ultrasound in the treatment of NRS, and the range of search time is January 1990 to January 2021. The searched literature is screened and evaluated by two researchers respectively according to the inclusion and exclusion criteria. If there is disagreement, discussing it with the third researcher to determine the final inclusion of the literature. Using the RevMan 5.3 software to conduct the meta-analysis.

**Results:**

This study will compare the effectiveness and safety of traditional Chinese medicine acupoint catgut embedding guided by musculoskeletal ultrasound in the treatment of NRS.

**Conclusion:**

The results of this study will be published in internationally influential academic journals to provide evidence-based medical evidence for the clinical effectiveness and safety of traditional Chinese medicine acupoint catgut embedding in the treatment of NRS.

**Ethics and dissemination:**

This study does not involve specific patients, and all research data comes from publicly available professional literature, so an ethics committee is not required to conduct an ethical review and approval of the study.

**OSF registration number:**

DOI 10.17605/OSF.IO/Q492E.

## 1 Introduction

Nerve root sciatica (NRS) is a common orthopedic disease, which mainly refers to the radiation pain along the sciatic nerve distribution area, mainly in the buttocks, the back of the thigh, the posterolateral side of the leg, and the dorsolateral side of the foot. The cause of the disease is mainly caused by lumbar herniated disk, which is often caused by bending or strenuous activity.^[1]^ The clinical symptoms of the patient are lower back pain and waist stiffness. As the progress of disease, the pain worsens. The pain starts from the waist, buttocks, and hips, and continues to the back of the thigh, popliteal fossa, outer calf, and back of the foot. The disease usually occurs between 20 and 40 years of age, and the incidence rate is increasing year by year and is being younger. It has been reported that about 60% of patients with sciatica cause mild dysfunction due to untimely treatment and inappropriate methods, and many patients have a long course of illness and recurrent onset.^[2]^ Long-term pain and dysfunction directly affect the normal life and work of patients, so the choice of appropriate and effective treatment for the disease has attracted wide attention from the relevant people in the medical profession.^[3]^

At present, Western medicine clinically believes that the patients with NRS have chronic inflammation in the vertebral tube, resulting in pain, so the treatment should improve the neuroinflammatory reaction and autoimmune reaction inflammation. These include pharmacotherapy, physiotherapy, traction therapy, nerve block therapy, and surgical therapy. Pharmacotherapy is a common way of the disease, the choice of drugs often uses painkillers, supplemented by sedatives and vitamins, and other nutrients to relieve edema. Adrenal cortical hormone and other hormone drugs can also be applied, but it often difficult to accept for the long-term by patients due to the side effects and high costs.^[4]^ The chemical radicular neuritis and autoimmune reaction mechanism of NRS caused by lumbar herniated disk has been confirmed. A large number of clinical trials have shown that 80% to 90% of patients with NRS can obtain more satisfactory relief by choosing non-surgical therapy, so surgical treatment is not a priority treatment.^[5–7]^ Nerve block therapy is also widely used at this stage. It is to inject medical ozone or corticosteroids and other drugs into the peripheral nerves of patients through injection, which can effectively relieve inflammatory reactions and block the malignant conduction of pain. Although it has achieved a certain application value, the effect is not significant, and long-term medication has a high recurrence rate.^[8]^ With the continuous development of traditional Chinese medicine technology, Chinese medicine believes that sciatica is mainly due to the evil of rheumatism and humidity, resulting in meridian damage, gas, and blood siltation, not general pain.^[9]^ At the same time, Chinese medicine treatment methods (including acupoint catgut embedding, needle knife, needle punching, massage techniques, etc) have achieved good results in the process of clinical application, which can quickly improve clinical symptoms, relieve pain, and it has the advantages of low cost, patient compliance, and protection of prognosis and others.^[10]^ Acupoint embedding is a method of inserting absorbable surgical sutures into acupoints and using the continuous stimulating effect of the thread on the acupoints to prevent and treat diseases. It plays an irreplaceable role in the treatment of orthopedic pain diseases. However, some problems and deficiencies have gradually been exposed during the clinical operation, which are mainly manifested in the lack of precision in the operation process and the lack of quantitative analysis of the depth of thread embedding. In recent years, with the advent of the era of medical big data, the rapid development of acupuncture imaging has opened the door for further exploration of quantitative acupoint embedding. As an important part of imaging, musculoskeletal ultrasound is widely used in the field of acupuncture and moxibustion. The examination has the characteristics of safety, non-invasiveness, no radiation damage, convenient and cheap, and reproducible examination in a short time. It can perform real-time dynamic observation in the movement of neurovascular muscles and tendons, effectively showing the anatomical features and positions of different tissues under the skin. Observe the hierarchical structure characteristics of the local cross-section of the acupoints, and accurately locate and qualitatively identify the diseased tissue. Therefore, musculoskeletal ultrasound provides great help to the accuracy of acupoint embedding. However, there is a lack of evidence of evidence-based medicine, so taking into account the above factors, this study uses systematic evaluation to conduct the scientific evaluation of the clinical effectiveness and safety of traditional Chinese medicine acupoint catgut embedding in the treatment of NRS, and provide evidence-based medical evidence support for the treatment of NRS.

## 2 Methods

### 2.1 Protocol register

This protocol has been registered on the Open Science Framework (OSF), registration number: DOI 10.17605/OSF.IO/Q492E. (https://osf.io/q492e).

### 2.2 Eligibility criteria

#### 2.2.1 Type of study

A randomized controlled study on acupoint catgut embedding guided by musculoskeletal ultrasound in the treatment of NRS. The language of the study is Chinese or English.

#### 2.2.2 Population

Clinically diagnosed patients with unilateral or bilateral lower limb radiation pain admitted to hospital, some patients may have sensory disorders, such as unilateral or bilateral lower limb numbness in varying degrees, perineal area sensory abnormalities are also common symptoms of a few patients, there is obvious tenderness beside the spinous process of lumbar vertebrae, percussion lumbar vertebra can appear radiation pain of lower limb, the straight leg elevation test is usually positive, and the sciatic nerve can be stretched to show positive signs. Computed tomography (CT) or magnetic resonance imaging (MRI) examination confirms the presence of one or more intervertebral disc herniation, resulting in the vertebral gap is narrow or the nerve root is crushed.

#### 2.2.3 Inclusion criteria

(1) Meet the diagnostic criteria of lumbar herniated disk in the Diagnosis and Curative Effect Criteria for Diseases and Syndromes of Traditional Chinese Medicine.
(2) Pain and numbness in the waist, buttocks, back of the thigh, posterior side of the calf, and outside of the foot, which are aggravated by coughing, sneezing, and exertion.
(3) Hyperesthesia or dullness in the innervated area of the lower limbs, the elderly with the course of the disease may see muscle weakness, muscle atrophy, the straight leg elevation test, and strengthening test are positive, the knee-tendon and achilles tendon reflex are weakened, and the back extension of the thumb is weakened.
(4) Lumbar CT or MRI clearly shows nerve root compression caused by the lumbar herniated disk.^[11]^
(5) Electromyogram examination shows that the sensory or motor nerve conduction velocity slows down.
(6) It is suitable for patients with non-surgical treatment.

#### 2.2.4 Exclusion criteria

(1) It does not conform to the diagnostic criteria of traditional Chinese medicine and Western medicine.
(2) The age is less than 18 years old and more than 75 years old.
(3) Combined with lumbar tumor, tuberculosis, fracture.
(4) Patients with central lumbar herniated disk and cauda equina compression.
(5) Patients with severe heart, lung, liver, kidney diseases, and psychosis.
(6) The use of hormones and anti-rheumatic drugs.
(7) Review, case study, animal experiment, and other non-randomized controlled studies.

#### 2.2.5 Types of interventions

**Figure 1:**
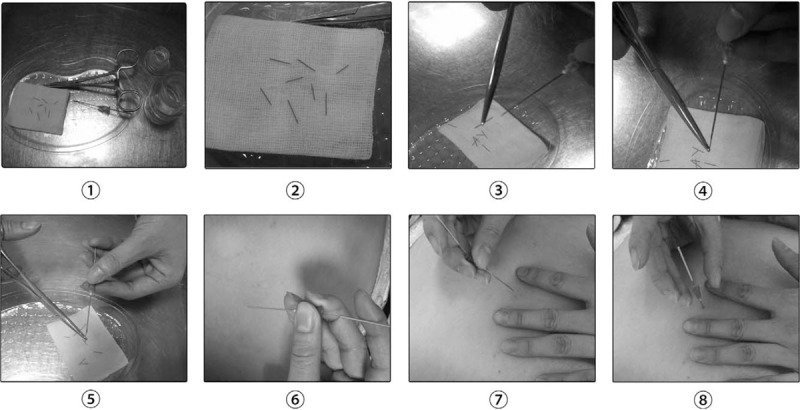
The operation steps of acupoint catgut embedding therapy.

Experimental interventions: Chinese medicine acupoint catgut embedding guided by musculoskeletal ultrasound. There is no limit to the age, weight, acupoint selection, and depth of catgut embedding in the 2 groups. Figure 1 shows the operation steps of acupoint catgut embedding therapy.

Control interventions: Western medicine conservative treatment, including nerve block therapy, physiotherapy, traction therapy, blank control, and so on (excluding oral and surgical treatment of drugs). There is no limit to the age, weight, and course of treatment in the 2 groups.

#### 2.2.6 Outcome indicators

The primary outcome indicators include:

(1) Total effective rate: Healing: the patient's symptoms of lower back and leg pain disappeared, and the straight leg raised more than 70°, and normal work is restored. Significant effect: the patient's symptoms of lower back and leg pain have basically disappeared, and the straight leg raised to 50° to 70°, and after rest, it can work later. Effective: the patient's symptoms of lower back and leg pain are significantly reduced, and the straight leg is raised up to 30° to 70° and it cannot return to normal work. Ineffective: no improvement in pain symptoms and signs. Total effective rate = healing rate + significant rate + effective rate.
(2) Using visual analog scale to assess the degree of pain, that is, a 10 cm long segment is divided into 10 parts, one end marked “painless,” the other end marked “most painful,” 0 points for painless, 10 points for the most painful, and finally let the patient mark the location of the line segment to indicate the degree of pain.^[12]^
(3) The Japanese Orthopaedic Association score, which consists of subjective symptoms, clinical signs, daily life restriction, and bladder function, and it is mainly used for the effectiveness evaluation of cervical and lumbar vertebral disorders after surgery, with a total score of 29 points, the higher the score indicates the better lumbar function.^[13]^
(4) The Oswestry disability index scale is used to evaluate the daily activities of patients with NRS caused by low back and leg pain. And it is divided into pain level, daily self-care ability, pick-up, walking, sitting, standing, sleep, sex life, social activities, travel (outings), and other 10 dimensions, a total score of 50 points, the higher the score indicates the more severe the dysfunction.^[14]^

The secondary outcome indicators include:

(1) The detection of nerve conduction velocity.
(2) The rate of adverse reactions.
(3) The cost of treatment.

### 2.3 Data sources and search strategies

It mainly adopts the method of computer retrieval in CBM, VIP, CNKI, WANFANG Database, PubMed, ScienceDirect, Web of Science, Embase, Cochrane Library, and searching the randomized controlled study on the clinical effectiveness and safety of traditional Chinese medicine acupoint catgut embedding guided by musculoskeletal ultrasound in the treatment of NRS; the range of retrieval time is from January 1990 to January 2021. Using the combination of mesh medical subject words and item words, the search terms are combined for the literature retrieval through the logical characters “OR” and “AND.” The range of retrieval is the title and abstract. Manual retrieval of all detected reviews, meta-analysis, and references included in the research literature is conducted to improve the recall rate of the literature. The results of the literature retrieval in Embase by subject words and free words are shown in Table 1.

**Table 1:** Results of the literature retrieval in Embase by subject words and free words.

Number	Search items
#1	’radicular sciatica’:ab,ti OR ’root sciatica’:ab,ti OR 'sciatica of nerve roots’:ab,ti OR ’nerve root sciacca’:ab,ti
#2	’acupoint embedding thread’:ab,ti OR ’catgut embedding at acupoints’:ab,ti OR ’acupoint embedding’:ab,ti OR ’point embedding therap’:ab,ti OR catgut:ab,ti OR ’medicated threads’:ab,ti
#3	’conservative treatment’:ab,ti OR ’conservative treatments’:ab,ti OR ’treatment, conservative’:ab,ti OR ’treatments, conservative’:ab,ti OR ’conservative management’:ab,ti OR ’conservative managements’:ab,ti OR ’management, conservative’:ab,ti OR ’managements, conservative’:ab,ti OR ’conservative therapy’:ab,ti OR ’conservative therapies’:ab,ti OR ’therapies, conservative’:ab,ti OR ’therapy, conservative’:ab,ti
#4	’physical therapy modalities’:ab,ti OR ’modalities, physical therapy’:ab,ti OR ’modality, physical therapy’:ab,ti OR ’physical therapy modality’:ab,ti OR ’physiotherapy, techniques’:ab,ti) OR ’physiotherapies, techniques’:ab,ti OR ’physical therapy techniques’:ab,ti OR ’physical therapy technique’:ab,ti OR ’techniques, physical therapy’:ab,ti OR ’group physiotherapy’:ab,ti OR ’group physiotherapies’:ab,ti OR ’physiotherapies, group’:ab,ti OR ’physiotherapy, group’:ab,ti OR ’physical therapy’:ab,ti OR ’physical therapies’:ab,ti OR ’neurological physiotherapy’:ab,ti OR ’therapy, physical’:ab,ti OR ’physiotherapy, neurological’:ab,ti OR neurophysiotherapy:ab,ti
#5	’musculoskeletal ultrasound’:ab,ti OR ’ultrasonic therapy’:ab,ti OR ’therapy, ultrasonic’:ab,ti OR ’therapies, ultrasonic’:ab,ti OR ’ultrasonic therapies’:ab,ti
#6	’phototherapy’:ab,ti OR ’phototherapies’:ab,ti OR ’therapy, photoradiation’:ab,ti OR ’photoradiation therapies’:ab,ti OR ’therapies, photoradiation’:ab,ti OR ’light therapy’:ab,ti OR ’light therapies’:ab,ti OR ’therapies, light’:ab,ti OR ’therapy, light’:ab,ti OR ’photoradiation therapy’:ab,ti
#7	’hydrotherapy’:ab,ti OR hydrotherapies:ab,ti OR ’whirlpool baths’:ab,ti OR ’bath, whirlpool’:ab,ti OR ’baths, whirlpool’:ab,ti OR ’whirlpool bath’:ab,ti
#8	’electric stimulation therapy’:ab,ti OR ’therapeutic electrical stimulation’:ab,ti OR ’electrical stimulation, therapeutic’:ab,ti OR 'stimulation, therapeutic electrical’:ab,ti OR ’therapeutic electric stimulation’:ab,ti OR ’electric stimulation, therapeutic’:ab,ti OR 'stimulation, therapeutic electric’:ab,ti OR ’electrical stimulation therapy’:ab,ti OR 'stimulation therapy, electrical’:ab,ti OR ’therapy, electrical stimulation’:ab,ti OR ’therapy, electric stimulation’:ab,ti OR 'stimulation therapy, electric’:ab,ti OR electrotherapy:ab,ti OR ’interferential current electrotherapy’:ab,ti OR ’electrotherapy, interferential current’:ab,ti
#9	’cryotherapy’:ab,ti OR ’cryotherapies’:ab,ti OR ’cold therapy’:ab,ti OR ’cold therapies’:ab,ti OR ’therapies, cold’:ab,ti OR ’therapy, cold’:ab,ti
#10	’hyperthermia, induced’:ab,ti OR ’therapy, fever’:ab,ti OR ’hyperthermia, therapeutic’:ab,ti OR ’induced hyperthermia’:ab,ti OR ’therapeutic hyperthermia’:ab,ti OR thermotherapy:ab,ti OR ’fever therapy’:ab,ti OR ’hyperthermia, local’:ab,ti OR ’local hyperthermia’:ab,ti
#11	#3 OR #4 OR #5 OR #6 OR #7 OR #8 OR #9 OR #10
#12	#1 AND #2 AND #11

### 2.4 Data collection and analysis

**Figure 2:**
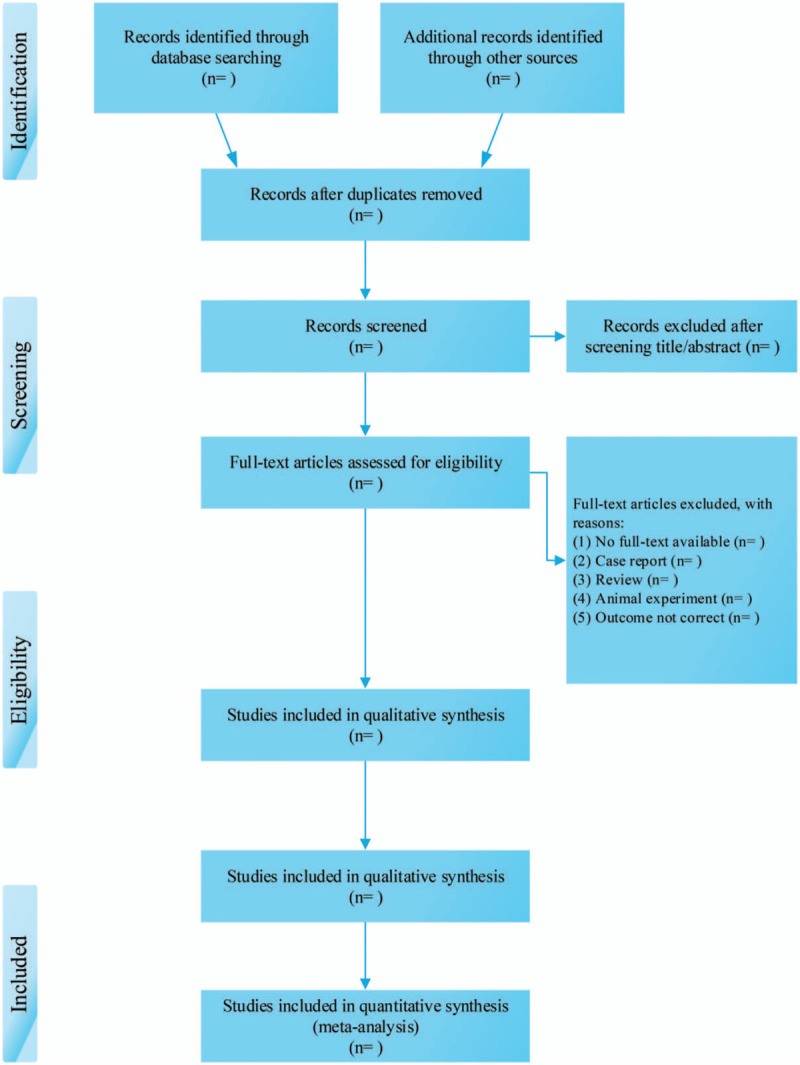
The documents retrieval process for Chinese and foreign language databases.

The two researchers independently carry out the literature screening. After retrieving the major databases by computer, all the filtered literature titles and abstracts are imported into the literature management software, duplicate literature is screened, literature titles, and abstracts are screened for literature that does not meet the inclusion criteria, and finally, the literature that meets the criteria is selected by reading the full text. The retrieval process is shown in Figure 2. If there are differences in the process of the literature screening, consulting the relevant experts or discussing them with a third researcher. If several papers have been published in the same study, the literature with the most complete experimental data and the most consistent with the inclusion criteria are selected for inclusion in the study. All the selected literature that met the inclusion criteria are sorted out and analyzed by office software: basic literature information (title, author, year of publication), general situation (patient age, gender, number of cases, etc.), experimental design (interventions), and outcome indicators. If the outcome indicator units in the literature are not consistent, the units are converted uniformly and then subsequent data processing is carried out. If there is a lack of data in the literature, trying to contact the original author by e-mail or telephone and obtain the relevant information needed. The documents retrieval process for Chinese and foreign language databases is shown in Figure 1.

### 2.5 Quality assessment

The selected literature conducts bias risk evaluation based on the method recommended by Coachrane Handbook for System Reviews of Interventions 5.1.0 and its results are reported. Evaluation items include random grouping methods, allocation of hidden scheme design, use of blind methods, reporting of results data, whether there is any selective reporting of research results, whether there are other sources of bias, and so on. The results are as follows: “Yes” represents that the method is correct or the data is complete, indicating that bias risk is small; “Unclear” represents that the method is not clear, indicating that bias risk is moderate; “No” represents that the method is incorrect or the data is incomplete, indicating that bias risk s is high. Finally, the evaluation results were input into RevMan 5.3 software, and the bias risk assessment chart is output.

### 2.6 Statistical method

Conducting statistical analysis using the RevMan 5.3 software recommended by the Cochrane Collaboration Network. The statistics of counting data are expressed by odds ratio, while statistics of measurement data are expressed by mean difference. Both the effect quantity of counting data and the measurement data are expressed as 95% confidence interval. For heterogeneity test, when the statistics *P* > 0.1, *I*^2^ < 50% can be considered to have a higher homogeneity between the results of the study, indicating that there is no significant statistical difference in the inclusion data, at this time, using the fixed-effect model; when the statistic *P* ≤ 0.1, *I*^2^ ≥ 50% can be considered that there is heterogeneity between the results of the study, indicating that there are significant statistical differences in the inclusion data, and taking into account the factors that might cause heterogeneity, and that there might be heterogeneity. The factors of increased quality are analyzed in subgroups according to actual needs, and if subgroup analysis shows *P* ≤ 0.1, *I*^2^ ≥ 50%, the results are that there is heterogeneity, using random effect models. On the contrary, if there are statistical differences in the results of each subgroup, but there is no clinical heterogeneity, using the fixed-effect model to conduct the meta-analysis when the heterogeneity of the outcome index is too large, it can be considered that the actual need for descriptive analysis of the relevant literature research. Sensitivity analysis is used to test whether the results are stable.

### 2.7 Sensitivity analysis

Sensitivity analysis: the implementation method is divided into change analysis model, one by one to remove the included research (deleting one by one a result indicator included in the study, observing whether the heterogeneity has changed while recording the numerical changes of the combined effect value. If it is found that there is a significant change in the heterogeneity of the outcome indicator after the removal of a document, then the literature is the source of heterogeneity). When the conclusions are unified before and after the sensitivity analysis, the conclusions are stable, and when the conclusions are inconsistent before and after the sensitivity analysis, the conclusions are not stable and it is needed to be treated with caution.^[15]^

### 2.8 Subgroup analysis

If necessary, according to the heterogeneity of outcome indicators, this study will conduct subgroup analysis from the selected acupoints, the depth of catgut embedding, the type of intervention, and the course of treatment in the control group, to find the source of heterogeneity and reduce the heterogeneity.

### 2.9 Publication bias

Publication bias is identified by funnel plot. Funnel plot is a qualitative evaluation method, which can be visually identified by funnel pattern symmetry to determine whether the bias exists. The included literature uses a funnel chart for publication bias analysis, if the two sides of the graph are basically symmetrical, then the bias is smaller, and conversely, the bias is larger.

### 2.10 Grading the quality of evidence

Based on the results of systematic evaluation, the quality of evidence is evaluated by GRADE, which is an internationally used evidence quality grading system, and the quality of evidence is graded as follows: (1) high quality: further research is unlikely to change the credibility of the results of the effectiveness assessment. (2) Medium quality: further research is likely to affect the credibility of the results of the effectiveness assessment and it may change the results. (3) Low quality: further research is likely to affect the credibility of the results of the effectiveness assessment, and the results are likely to change. (4) Very low quality: the results of any effectiveness assessment are uncertain. Recommendations are classified as “Strong” and “Weak”: strong recommendations indicate that the evaluator has a clear indication that the intervention has more advantages or disadvantages than benefits, and weak recommendations indicate that the intervention has more advantages or disadvantages than benefits. The included study is all randomized controlled trials, and although evidence is first classified as high quality based on randomized controlled trials, the quality of such evidence may be reduced by five factors: risk of research bias, discrete research results, indirect evidence, imprecise results, and publication bias.

## 3 Discussion

With the change of people's lifestyle and the coming of aging society in modern society, the incidence of NRS caused by the lumbar herniated disk is on the rise. NRS is a clinically common secondary nerve pain, which is mainly caused by the pressure of peripheral tissue or lesions, mostly in stooping, force or intense activity acute or subacute disease, a few patients for chronic oncology.^[16]^ The lesions are located in the vertebral tube, through imaging examination can be cleared for a lumbar herniated disk, bulging, lumbar osteoarthritis, hypertrophic spondylitis, and lumbar spinal stenosis. The patient is manifested persistent or paroxysmal pain along the sciatic nerve pathway and its distribution area. The incidence is mostly single-sided and accompanied by burning or knife-like pain, especially at night, seriously affecting the life quality of patients. At present, the specific pathogenesis of the disease is not clear. Most experts believe that the abnormal discharge of nerve root electrophysiology is directly related to the radiation pain of the sciatic nerve, in which physical oppression, pulling, and chemical inflammatory factor stimulation are important causes of nerve root electrophysiological abnormalities.^[17]^ Generally speaking, the NRS patients have a wide range of pain, and radioactive pain is the main manifestation, clinical treatment methods are diverse. Nerve block therapy is one of them, which is a method of injecting medical ozone or drugs into the peripheral nerve to relieve or eliminate pain by eliminating nerve inflammation and blocking the pain conduction pathway. Injection therapy is commonly combined with medical ozone, corticosteroids, low concentration local anesthetics, B vitamins, and so on, of which medical ozone, corticosteroids are the main drugs. Because injection therapy can directly target medical ozone and corticosteroids to the inflammatory site, forming a local higher concentration, so it can significantly reduce and eliminate inflammatory reactions. However, the disadvantage is poor effectiveness and a high recurrence rate.^[18]^

Sciatica is classified as the category of paralysis and back and leg pain in motherland medicine. As a common clinical disease, it is recorded a lot in ancient Chinese medicine literature. The etiology, pathogenesis, syndrome differentiation, and treatment methods of the disease have been continuously inherited and developed in the past dynasties, and the theoretical basis and practical experience have been constantly improved. Among them, Chinese medicine acupoint catgut embedding as one of the treatment methods, which is based on acupuncture therapy and developed a combination of Chinese and Western medicine treatment methods. Acupoint catgut embedding method puts absorbable surgical suture or catgut soaked in traditional Chinese medicine into corresponding acupoints through catgut embedding needle, and it can absorb surgical stitches or sheep intestines decomposition process generally lasts 1 to 2 weeks. In the process of decomposition of the acuity to produce mild and sustained benign stimulation, enhancing the amount of stimulation to the point, regulating the body environment, and increasing the body repair and resistance. In addition, with the insertion of the embedding needle, the local adhesive tissue can be released and blood stasis can be induced, so as to improve local circulation, eliminate nerve root edema and improve pain symptoms.^[19]^ In general, the acupoint catgut embedding method makes up for the shortcomings of ordinary acupuncture or traditional electroacupuncture, such as short time, long course of treatment and repeated acupuncture, prolongs the needle retention time, and strengthens the therapeutic effect. On the one hand, it can activate blood circulation, regulate blood, and on the other hand, it can promote the repair function of body tissue. To achieve the therapeutic purposes, so as to balance and coordinate the meridians, viscera, and limbs of the human body. Some scholars also pointed out that catgut embedding therapy can enhance immunity, play a benign induction role, and the operation of acupoint catgut embedding method is simple and safe, which can reduce the number of patient visits, enhance patient compliance. At the same time, combined with musculoskeletal ultrasound guidance, it can help diagnose small liquid soft tissue collections, and can display the nerves and blood vessels and the distance from the skin during the scan. Precise positioning by ultrasound before embedding, precise acupoint embedding under the guidance of musculoskeletal ultrasound, you can observe the position and depth of the embedding needle and the tissue structure of adjacent activities, so you can adjust the direction and angle in time to avoid misalignment Damage to vital organs and tissues. This method can expand the scope of clinical acupoint selection, thereby further expanding the scope of acupoint embedding therapy to treat diseases. However, there is a lack of evidence of evidence-based medicine, so taking into account the above factors, this study uses systematic evaluation to conduct the scientific evaluation of the clinical effectiveness and safety of traditional Chinese medicine acupoint catgut embedding in the treatment of NRS, and provide evidence-based medical evidence support for the treatment of NRS.

## Author contributions

**Conceptualization:** Junqing Wang, Zhaoyi Han.

**Data curation:** Zhaoyi Han, Xiaowei Li.

**Formal analysis:** Zhaoyi Han, Xiaowei Li, Zhi Liu.

**Funding acquisition:** Junqing Wang, Junqing Wang.

**Resources:** Zhaoyi Han, Xiaowei Li, Zhi Liu, Morigen Bai.

**Software:** Zhaoyi Han, Xiaowei Li, Zhi Liu, Zhihui Zhao.

**Supervision:** Zhaoyi Han, Zhi Liu.

**Writing – original draft:** Zhaoyi Han, Xiaowei Li, Zhi Liu, Morigen Bai, Zhihui Zhao.

**Writing – review & editing:** Junqing Wang.
